# Artificial Sweeteners and Pancreatic Cancer: Is Aspartame a Culprit or a Coincidence?

**DOI:** 10.12669/pjms.40.4.8490

**Published:** 2024

**Authors:** Sanghoon Han, Jieun Yang, Ji Eun Park, Jung Ho Kim

**Affiliations:** 1 Sanghoon Han, Department of Internal Medicine, Jeju National University College of Medicine, Jeju National University Hospital, Jeju, Republic of Korea; 2 Jieun Yang, Department of Internal Medicine, Jeju National University College of Medicine, Jeju National University Hospital, Jeju, Republic of Korea; 3 Ji Eun Park, Department of Internal Medicine, Jeju National University College of Medicine, Jeju National University Hospital, Jeju, Republic of Korea; 4 Jung Ho Kim Department of Internal Medicine, Gachon University Gil Medical Center, Gachon University College of Medicine, Gachon University

**Keywords:** Artificial Sweeteners, Pancreatic Cancer, Aspartame, Carcinogenicity, Cancer Risk

Aspartame’s designation by the International Agency for Research on Cancer as “possibly carcinogenic to humans” has raised questions about its potential association with pancreatic cancer. By requesting further investigations to ascertain the veracity of this relationship, this study seeks to contribute to this ongoing debate. Understanding the effect of aspartame on cancer risk is essential for public health, although the research remains ambiguous.

A case-control study (doi: 10.1158/1055-9965. EPI-09-0365) was conducted in 2009 by Bosetti et al. to determine whether low-calorie sweeteners such as aspartame increase the risk of developing gastric, pancreatic, and endometrial malignancies. Many participants were evaluated between 1991 and 2014. This significant pancreatic cancer trial involved 326 patients and 652 controls. The consumption of low-calorie sweeteners and the risk of several diseases, including pancreatic cancer, were not significantly correlated, according to the study.

In 2017, one study investigated the effects of artificial sweeteners, such as aspartame and stevia, on pancreatic acinar cancer. A mouse model was used in this study. In a previous study, aspartame and stevia did not increase the incidence of pancreatic cancer in a mouse model.^1^

However, a study conducted in 2021 found that long-term aspartame exposure enhanced the number of cancer stem cells (CSC) and aggressiveness of tumour cells in pancreatic cancer cells.^2^ Additional research is required to fully comprehend the connection between artificial sweeteners and the risk of developing cancer.

It is important to remember that a recent study from 2023 examined the health advantages of using Delonix regia extract as a natural substitute for artificial sweeteners. The antioxidant and antibacterial capabilities of the extract and its capacity to slow the growth of pancreatic cancer cells have been demonstrated in a previous study.^3^ The findings of this study show that it is not reasonable to conclude that all artificial sweeteners, and not just aspartame, may pose a risk for cancer.

The link between aspartame and cancer, particularly pancreatic cancer, is convoluted. Existing research provides essential insights, but there is a dearth of investigations. Future research must take into account dose, exposure duration, and combinations of factors to provide clear evidence and recommend the use of artificial sweeteners.

## Author’s Contribution:

**SH:** Is the first author and proposed the idea of the study.

**SH**, **JY**, **JP** and **JK:** Researched the literature and wrote the manuscript.

**JK:** Reviewed the literature.
